# Evaluation of Current Tarnished Plant Bug (Hemiptera: Miridae) Thresholds in Transgenic MON 88702 Cotton Expressing the Bt Cry51Aa2.834_16 Trait

**DOI:** 10.1093/jee/toaa075

**Published:** 2020-04-25

**Authors:** J C Corbin, T B Towles, W D Crow, A L Catchot, D R Cook, D M Dodds, J Gore

**Affiliations:** 1 Department of Biochemistry, Molecular Biology, Entomology, and Plant Pathology, Mississippi State University, Mississippi State, MS; 2 Delta Research and Extension Center, Mississippi State University, Stoneville, MS; 3 Department of Plant and Soil Science, Mississippi State University, Mississippi State, MS

**Keywords:** IPM, lygus resistant, *Bacillus thuringiensis*

## Abstract

The tarnished plant bug, *Lygus lineolaris* (Palisot de Beauvois), is an important pest of cotton in many areas of the southern United States. An experiment was conducted at two locations in Mississippi during 2016 and 2017 to evaluate action thresholds for tarnished plant bug on a novel *Bacillus thuringiensis* cotton that expresses the Cry51Aa2.834_16 toxin. Treatments included the current action threshold, a 2× threshold, and treatments where insecticides were only applied during the early season (preflower) or only during late season (during flowering) based on the current action thresholds. These were compared to an untreated control and a weekly insecticide use regime that received weekly insecticide sprays. All treatments were imposed on both Bt Cry1Aa2.834_16 cotton and a nontraited cotton. The Bt Cry1Aa2.834_16 trait reduced the number of tarnished plant bugs and injury, and improved yields compared to nontraited cotton. For all spray treatments except the weekly insecticide use regime, yields were greater for the Bt Cry51Aa2.834_16 cotton than the nontraited cotton. In terms of thresholds, Bt Cry1Aa2.834_16 cotton sprayed based on current action thresholds resulted in similar yields to the weekly insecticide use regime of both cotton types. In contrast, the 2× threshold resulted in lower yields than the current threshold for both cotton types. Though thresholds intermediate to the currently recommended action threshold and the 2× threshold were not tested, these data suggest that currently recommended action thresholds appear appropriate for Bt Cry51Aa2.834_16 cotton. These results suggest that this trait will be an important component of current IPM programs in cotton where tarnished plant bug is an important pest.

The tarnished plant bug, *Lygus lineolaris* (Palisot de Beauvois), is the most economically damaging insect pest of cotton in the Mid-South region of the United States including the states of Arkansas, Mississippi, Louisiana, Tennessee, and Missouri (Musser et al. 2007, Gore et al. 2012). Tarnished plant bug resistance to numerous insecticide classes has become prevalent across the Mid-South (Snodgrass 1994, 1996; Snodgrass et al. 2009). Cook (2018) reported that 2.9–5.0 insecticide applications were necessary to prevent economic losses in the Mid-South during the 2017 season. Increasing costs for tarnished plant bug management with foliar insecticides, in addition to other input costs such as technology fees associated with the purchase of transgenic seed varieties, increased fuel and fertilizer costs, and increased herbicide use due to resistant weed species, as well as control costs for other insect pests in cotton (Riley et al. 2010), have led to a decline in cotton production across the Mid-South.

Tarnished plant bug can cause damage to cotton at any growth stage, with most of the economic damage occurring from first-square until early bloom (Scales and Furr 1968). Tarnished plant bug populations exceeding current action thresholds in early season cotton can result in reduced plant height and boll weight, as well as swollen nodes, deformed leaves, and delayed maturity (Scales and Furr 1968, Hanny et al. 1977). Cotton squares less than 3.18 mm in diameter are preferred feeding sites for tarnished plant bug as opposed to bolls and larger squares (Tugwell et al. 1976). Feeding on small cotton squares often results in abscission of those squares (Layton 1995) which can result in an altered fruiting pattern if early square loss is excessive. A single tarnished plant bug can cause the abscission of 0.6–2.1 squares per day (Gutierrez et al. 1977). Tarnished plant bug will also feed on larger squares, which may also abscise, but generally remain on the plant and produce a bloom depending on the severity of the feeding damage. This damage will be apparent on cotton blooms as the anthers will be dark brown in color. When ≤30% of anthers are damaged there is little to no yield loss, compared to an increase in malformed bolls, boll abscission, and subsequent yield loss when anther damage is >30% (Pack and Tugwell 1976). Boll loss and malformed bolls is likely the result of poor pollination due to damaged anthers caused by tarnished plant bug feeding. Tarnished plant bug also causes direct damage by feeding on bolls, causing sunken lesions on the outside of the boll that eventually turns black and necrotic (Pack and Tugwell 1976). Tarnished plant bug feeding damage on larger, more developed bolls is not as common, but can result in individual seed damage which results in discolored lint and reduced overall boll weight (Pack and Tugwell 1976).

Until recently, *Bt* cotton has been confined to the control of lepidopteran pests in cotton. Bayer Company is currently developing a transgenic event for cotton (MON 88702) that expresses the Cry51Aa2.834_16 protein from *Bacillus thuringienses* that targets hemipteran and thysanopteran insect pests, particularly *Lygus* spp. and thrips, *Frankliniella* spp. (Bachman et al. 2017). This new Bt event is being developed with hopes of reducing insecticide control costs for tarnished plant bug in cotton. MON 88702 should also extend the useful life of other insecticides by reducing the exposure of tarnished plant bug to current insecticides, resulting in a decreased rate of selection for resistance. MON 88702 is not expected to be 100% effective on tarnished plant bug, but previous research showed a reduction in the number of sprays needed for tarnished plant bug in cotton when sprayed at the current action threshold (Graham and Stewart 2018). Research is needed to determine at what population density this event provides effective control of tarnished plant bug without sustaining economic loss. Therefore, research was conducted as an initial attempt to evaluate the currently recommended action threshold for tarnished plant bug on MON 88702 cotton expressing the Bt Cry51Aa2.834_16 protein.

## Materials and Methods

An experiment was conducted at the Delta Research and Extension Center in Stoneville, MS and on Sidon Plantation in Sidon, MS in 2016 and 2017 to determine the appropriate threshold for tarnished plant bug on cotton containing the MON 88702 gene. Trials were arranged in a randomized complete block design with a two by six factorial arrangement of treatments. Factor A was cotton variety and included MON 88702 that expresses the Cry51Aa2.834_16 protein from *Bacillus thuringiensis* versus a conventional cotton variety (Deltapine 393). Factor B consisted of threshold treatments which included an untreated control, weekly control, treatments applied using current Mississippi thresholds throughout the entire season, treatments applied using the current Mississippi thresholds from first square to first flower and then not treated the rest of the season (Early Season Control Only), treatments applied using the current Mississippi thresholds from first flower until cutout but not sprayed prior to first flower (Late Season Control Only), and treatments applied using 2× the current Mississippi thresholds throughout the season. The current square retention threshold in Mississippi is to treat when less than 80% of first position squares remain on the plant prior to first bloom (Catchot et al. 2018). The current Mississippi sweep net threshold is eight tarnished plant bugs per 100 sweeps during the first 2 wk of squaring and 15 tarnished plant bugs per 100 sweeps from the third week of squaring through bloom (Catchot et al. 2018). The current Mississippi drop cloth threshold is one tarnished plant bug per 1.5 row-m during the first 2 weeks of squaring and three tarnished plant bugs per 1.5 row-m from the third week of squaring through bloom (Catchot et al. 2018). The current Mississippi threshold for dirty squares is when 10% of the squares are stained, looking at medium-sized squares with exposed buds that have been discolored by plant bug feeding (Catchot et al. 2018). Sprays were made based on when any one of the thresholds (i.e., drop cloth numbers or dirty squares during bloom) was met or exceeded.

Planting dates during 2016 were 17 May in Sidon, MS and 23 May in Stoneville, MS. Both trials were planted on 17 May during 2017. Plots consisted of four 97-cm wide rows in Sidon, MS and four 102-cm wide rows in Stoneville, MS with 12.2-m length at both locations. Cotton was planted at 120,000 seed per hectare into raised conventional tilled beds. Seed were treated with a commercial premix of thiodicarb, imidacloprid, trifloxystrobin, triadimenol, and metalaxyl (Aeris Trilex Advanced, Bayer CropScience, Raleigh, NC) to minimize the impact of early season insect and disease pests. Pre-emergence and postemergence applications of herbicides were used for control of weeds. Planting depth was approximately 2.5-cm below the soil surface. Furrow irrigation was applied to the trial area as needed. Other insect pests were managed as needed with insecticides that do not have activity against tarnished plant bug. Two sequential applications of chlorantraniliprole (Prevathon, FMC Corporation, Princeton, NJ) were made 14 d apart starting at first flower to manage lepidopteran pests. No other pest species were observed in these experiments.

Plots were sampled twice per week throughout the growing season on the same days (Mon. and Thurs.) each week. Data collection methods for tarnished plant bug varied throughout the growing season depending on cotton growth stage. During the first 3 wk of squaring, square retention was recorded weekly in each plot. First position fruiting sites from the upper three nodes on 25 plants per plot were examined to determine square (flower bud) retention. The presence of an abscission scar at the first position on each fruiting branch was used as an indicator for a missing (abscised) square and assumed to be the result of tarnished plant bug feeding. Squares with evidence of tarnished plant bug damage such as blasted squares (very young squares on the top two nodes that have turned brown and abscise when touched) and squares with open (flared) bracts were also considered abscised and attributed to tarnished plant bug feeding. Also during the first 3 weeks of squaring, samples were taken with a 38-cm diameter sweep net twice per week. Fifteen sweeps per plot were taken, alternating sampling on row one and row four. During the flowering stages, all plots were sampled once per week with a 0.76-m black drop cloth. Two drop cloth samples were collected per plot. Samples were taken by positioning the drop cloth between the second and third rows near the center of the plot and vigorously shaking all of the cotton plants from each row onto the cloth so that 1.53-m of row were sampled for each drop. Tarnished plant bugs were separated into four categories: adults, small nymphs (first and second instar), medium nymphs (third and fourth instar), and large nymphs (fifth instar). Dirty square samples were also taken weekly during the flowering period, but were recorded on different days than the drop cloth samples. This was done so that the test could be sampled twice per week similar to what is currently recommended. In total, 25 randomly selected squares per plot were visually examined on plants (nondestructive) for evidence of tarnished plant bug feeding as described by Gore et al. (2012).

The untreated control plots were not sprayed for tarnished plant bug at any point during the growing season. The weekly control plots were sprayed once per week, regardless of tarnished plant bug population densities. All threshold treatments were sprayed the same day as sampling if the threshold for a particular treatment was exceeded. Thresholds were based on an average of all four replications within a treatment and included all life stages and sizes of tarnished plant bug. Insecticides utilized for control of tarnished plant bug included sulfoxaflor (Transform WG, Corteva Agricience, Indianapolis, IN), thiamethoxam (Centric 40 WG,Syngenta Crop Protection, Greensboro, NC), acephate (Orthene 90S,Valent Corporation, Walnut Creek, CA), and acephate plus bifenthrin (Brigade 2EC, FMC Corporation, Princeton, NJ). Individual decisions on which insecticides were used were based on previous insecticide use in the plot area and every attempt was made to ensure the maximum level of control with each spray. In all cases, the maximum labeled rate was used except in the case of sulfoxaflor and acephate when mixed with bifenthrin. Tarnished plant bug sampling was terminated when cotton reached five nodes above white flower (NAWF) plus 350–400 heat units (DD60s). To calculate nodes above white flower, main stem nodes were counted above the uppermost first position white flower (Bourland et al. 1992). Tarnished plant bug cannot damage cotton bolls once they have accumulated at least 300 heat units (Russell et al. 1999). Consequently, it can be assumed that the latest harvestable bolls are safe from tarnished plant damage when plants average five nodes above white flower (Bourland et al. 1992) plus 300 heat units (Russell et al. 1999).

At the end of the growing season, rows two and three of every plot were harvested mechanically with a cotton picker modified for small plot harvest and seedcotton weights were recorded. Lint yield was calculated using an average lint percentage of 39%. All in-season sampling data were analyzed as seasonal means across all sampling dates, locations, and years (total of 16 replications). All data were analyzed with a mixed model analysis of variance (PROC GLIMMIX, SAS version 9.3, Cary, NC). Trait and insecticide regime were considered fixed effects, while site-year, replication nested in site-year, replication by trait nested in site-year, and replication by spray nested in site-year were designated as random effects. Site-year was included in the random effects to allow inference over multiple years and locations (Blouin et al. 2011). For drop cloth samples, separate analyses were done for different life stages and sizes of the nymphs as well as total number of tarnished plant bugs. The test at Sidon, MS in 2017 had mechanical problems with the cotton picker and could not be accurately harvested, so those yields were not included in the analysis. For the analysis of preflower tarnished plant bug densities (sweep net) and percent square retention, the early season only treatment was pooled with the threshold treatment because those treatments were identical to each other at that point in the season (both sprayed at the same threshold level). Similarly, tarnished plant bug densities and square retention data for the late season only treatment were pooled with data from the untreated plots because those treatments were identical at that point in the season (no preflower sprays in either treatment). The number of sprays were analyzed by spray treatment regime to compare the number of sprays for MON 88702 cotton to nontraited cotton for each spray regime. Because all four replications within a location and year were sprayed the same, locations served as replications (2 locations × 2 yr equals 4 replications) for this analysis. All insect count data (log_10_ × + 1) and percentage data (log_10_) were transformed to normalize their distribution prior to analysis (Zar 1999). A Gaussian distribution was used for all data based on model fit and data distribution criteria (Little et al. 1996). For all analyses, degrees of freedom were calculated using the Kenward-Roger method (Kenward and Roger 1997). Means and standard errors presented in tables and graphs were calculated using PROC MEANS. The LSMEANS statement was used to separate means based on Tukey’s HSD test (α = 0.05, Tukey 1953).

## Results and Discussion

### Pre-Flower Samples

During the preflowering stages of cotton development, trait (*F* = 2.32; df = 1, 16.3; *P* = 0.15) and the trait by spray regime interaction (*F* = 1.77; df = 3, 117.4; *P* = 0.16) did not impact tarnished plant bug numbers based on sweep net samples. Spray regime did impact tarnished plant bug numbers prior to first flower based on sweep net samples (*F* = 6.3; df = 3, 32.9; *P* < 0.01). All of the spray treatments resulted in fewer tarnished plant bugs than the untreated control (Table 1). Weekly means and standard errors for tarnished plant bug numbers based on sweep net samples during the preflowering period are presented in a supplementary table (Supp Table 1 [online only]). In terms of plant injury prior to first flower, trait (*F* = 4.19; df = 1, 29.2; *P* = 0.05) and spray regime (*F* = 17.69; df = 3, 41.1; *P* < 0.01) impacted seasonal mean percent square retention, but the interaction between those two factors did not (*F* = 1.90; df = 3, 111.9; *P* = 0.13). Seasonal mean percent square retention was greater in the MON 88702 cotton (87.4 ± 0.7) than cotton without the trait (81.8 ± 1.0) regardless of spray regime. Square retention was different between all spray regime treatments (Table 1). Square retention ranged from a mean (SEM) of 88.7% (1.2) in the weekly spray treatment to 81.3% (1.2) in the untreated control. Thresholds for square retention vary slightly among states. Insecticide sprays are recommended when square retention falls below 80% in Mississippi (Catchot et al. 2018) and Tennessee (Stewart and McClure 2019). The critical level varies from 70 to 85% depending on crop condition and time of year in Arkansas (Studebaker 2020) and Louisiana (Ring 2019). Based on these results, the 2× threshold did not maintain acceptable levels of square retention. Weekly means and standard errors for percent square retention during the preflowering period are presented in a supplementary table (Supp Table 2 [online only]).

**Table 1.** Impact of the main effect of threshold spray treatment on seasonal mean number of tarnished plant bugs per 25 sweeps and percent square retention during preflowering stages of cotton development averaged across two locations in Mississippi and averaged across 2016 and 2017

	Mean (SEM)^*a*^	
	No. per 25 Sweeps	Percent Square Retention
Weekly	0.79 (0.08)B	88.7 (1.2)A
Threshold	1.18 (0.08)B	86.1 (1.1)B
2× Threshold	1.39 (0.15)B	83.9 (1.7)C
Untreated	1.52 (0.13)A	81.3 (1.2)D

^*a*^Means within a column followed by the same letter are not significantly different according to Tukey’s HSD (α = 0.05).

### Flowering Samples

**Fig. 1.**
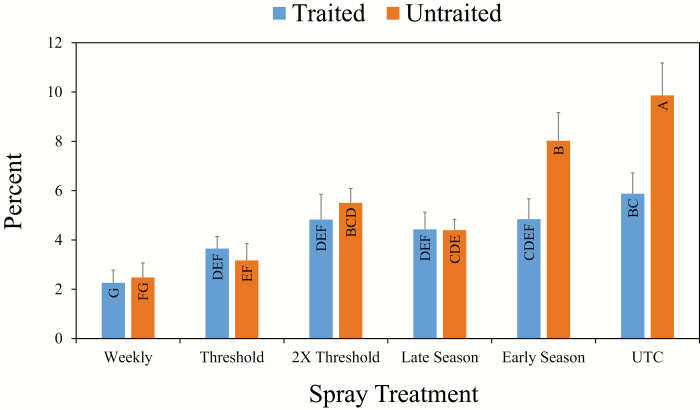
Impact of the cotton trait (Bt Cry51Aa2.834_16 vs. conventional) by threshold spray treatment interaction on seasonal mean (SEM) percentage of dirty squares averaged across two locations in Mississippi during 2016 and 2017. Means within the graph with the same letter are not significantly different according to Tukey’s HSD (α = 0.05).

During the flowering period, trait (*F* = 5.79; df = 1, 15; *P* = 0.02), spray regime (*F* = 10.72; df = 5, 58; *P* < 0.01), and the interaction between trait and spray regime (*F* = 2.64; df = 5, 75; *P* = 0.03) impacted the seasonal mean for percentage of dirty squares. No differences were observed between MON 88702 and nontraited cotton for the weekly, threshold, 2× threshold, and late season spray regimes (Fig. 1). Seasonal mean percentage of dirty squares was lower in MON 88702 cotton than nontraited cotton for the early season only and untreated spray regimes, the two spray regimes that were not sprayed during the flowering period. Weekly means and standard errors for percent dirty squares during the flowering period are presented in a supplementary table (Supp Table 3 [online only]).

The interaction between trait and spray regime impacted all life stages and sizes of tarnished plant bug except medium sized nymphs (Table 2) based on drop cloth samples during the flowering period. The MON 88702 cotton had fewer total tarnished plant bugs, adults, total nymphs, small nymphs, and large nymphs than the nontraited cotton for the threshold and untreated control spray regimes (Table 3). Differences between MON 88702 cotton and nontraited cotton were also observed for total tarnished plant bugs and total nymphs in the weekly spray regime. For the early season only and late season only spray regimes, differences were observed between MON 88702 cotton and nontraited cotton for adults and large nymphs, respectively (Table 3). Tarnished plant bug numbers were similar between MON 88702 cotton and nontraited cotton for the 2× threshold spray regime for all life stages and sizes. For MON 88702 cotton, differences were observed between the weekly spray regime and the threshold spray regime for total tarnished plant bugs and small nymphs, but no other life stages. In contrast, the current threshold treatment had more total tarnished plant bugs, and total, small, and large nymphs than the weekly spray regime for the nontraited cotton (Table 3). For medium-sized nymphs, trait and spray regime had an impact (Table 2). The MON 88702 cotton (0.95 ± 0.09) had fewer medium-sized nymphs than nontraited cotton (1.59 ± 0.14). The weekly spray regime had fewer medium nymphs than all other spray treatments (Table 3). Additionally, the current threshold and late season only spray regimes had fewer medium nymphs than the untreated control. In cotton that was not sprayed for tarnished plant bug (untreated), the MON 88702 cotton had 40.8, 51.5, 38.0, 33.2, and 44.0% fewer total tarnished plant bugs, adults, nymphs, small nymphs, and large nymphs than the nontraited cotton, respectively. In contrast, MON 88702 cotton had 61.9, 49.0, 63.6, 63.9, and 72.8% fewer tarnished plant bugs at those same life stages, respectively, than the nontraited cotton for the threshold spray regime. This suggests that insecticide sprays will be an important component of tarnished plant bug IPM and that MON 88702 cotton will complement current IPM programs for reducing tarnished plant bug numbers in cotton. Weekly means and standard errors for tarnished plant bug numbers based on drop cloth samples during the flowering period are presented in a supplementary table (Supp Table 4 [online only]).

**Table 2.** Analysis of variance for number of *Lygus lineolaris* life stages per 3.01-m of row based on drop cloth samples during flowering stages of cotton for and experiment evaluating MON 88702 cotton conducted at two locations in Mississippi during 2016 and 2017

	*F*-value, *P* > *F*		
Life Stage	Trait (df = 1, 15)	Spray (df = 5, 75)	Interaction (df = 5, 75)
**Total**	4.81, 0.04	44.61, <0.01	3.10, 0.01
**Adult**	5.66, 0.03	11.65, <0.01	2.55, 0.03
**Nymphs**	4.90, 0.04	42.44, <0.01	2.52, 0.04
**Small Nymphs**	3.16, 0.09	23.09, <0.01	2.29, 0.05
**Medium Nymphs**	7.59, 0.01	28.20, <0.01	1.11, 0.36
**Large Nymphs**	7.40, 0.01	19.45, <0.01	2.80, 0.02

**Table 3.** Impact of the interaction between MON 88702 cotton expressing the Bt Cry51Aa2.834_16 insecticidal protein and threshold spray treatments on seasonal mean numbers of *Lygus lineolaris* life stages and sizes per 3.01-m of row based on drop cloth samples during flowering stages of cotton for experiments conducted at two locations in Mississippi during 2016 and 2017

Total—Mean (SEM) per 3.01 m^*a*^						
	Weekly Spray	Threshold	2× Threshold	Late Season Only	Early Season Only	Untreated
**MON 88702**	0.95 (0.16)E	2.46 (0.30)D	6.46 (0.76)BC	4.64 (0.64)C	6.10 (1.02)BC	6.15 (0.88)BC
**Non-Bt**	2.32 (0.47)D	6.46 (1.39)BC	7.41 (0.70)B	5.36 (0.64)C	9.02 (1.44)BC	10.40 (1.00)A
**Adult—Mean (SEM) per 3.01 m** ^***a***^						
	**Weekly Spray**	**Threshold**	**2× Threshold**	**Late Season Only**	**Early Season Only**	**Untreated**
**MON 88702**	0.27 (0.07)F	0.4 (0.09)EF	0.73 (0.10)BCD	0.53 (0.09)CDE	0.72 (0.13)CD	0.8 (0.17)BCD
**Non-Bt**	0.49 (0.11)DEF	0.79 (0.15)BC	0.88 (0.14)BC	0.63 (0.12)CDE	1.31 (0.33)B	1.65 (0.23)A
**Nymph—Mean (SEM) per 3.01 m** ^***a***^						
	**Weekly Spray**	**Threshold**	**2× Threshold**	**Late Season Only**	**Early Season Only**	**Untreated**
**MON 88702**	0.68 (0.15)E	2.06 (0.27)D	5.73 (0.69)BC	4.12 (0.58)C	5.38 (0.97)BC	5.35 (0.78)BC
**Non-Bt**	1.83 (0.45)D	5.66 (1.35)C	6.53 (0.66)AB	4.74 (0.56)BC	7.72 (1.20)BC	8.74 (0.89)A
**Small Nymphs (first to second Instar)—Mean (SEM) per 3.01 m** ^***a***^						
	**Weekly Spray**	**Threshold**	**2× Threshold**	**Late Season Only**	**Early Season Only**	**Untreated**
**MON 88702**	0.51 (0.11)E	1.27 (0.22)D	3.58 (0.46)AB	2.93 (0.47)ABC	2.45 (0.48)C	2.55 (0.42)C
**Non-Bt**	1.13 (0.27)DE	3.52 (1.03)BC	4.19 (0.56)A	2.90 (0.40)ABC	3.03 (0.49)BC	3.82 (0.52)A
**Medium Nymphs (third to fourth Instar)—Mean (SEM) per 3.01 m** ^***a***^						
	**Weekly Spray**	**Threshold**	**2× Threshold**	**Late Season Only**	**Early Season Only**	**Untreated**
**MON 88702**	0.11 (0.05)	0.51 (0.11)	1.32 (0.24)	0.78 (0.14)	1.56 (0.26)	1.41 (0.21)
**Non-Bt**	0.49 (0.16)	1.12 (0.28)	1.63 (0.21)	1.04 (0.19)	2.87 (0.47)	2.42 (0.27)
**Mean**	0.30 (0.09)D	0.82 (0.16)C	1.48 (0.16)B	0.91 (0.12)C	2.21 (0.29)A	1.91 (0.19)AB
**Large Nymphs (fifth Instar)—Mean (SEM) per 3.01 m** ^***a***^						
	**Weekly Spray**	**Threshold**	**2× Threshold**	**Late Season Only**	**Early Season Only**	**Untreated**
**MON 88702**	0.06 (0.06)F	0.28 (0.06)EF	0.83 (0.15)C	0.40 (0.14)DE	1.37 (0.44)BC	1.40 (0.33)BC
**Non-Bt**	0.21 (0.07)EF	1.03 (0.29)C	0.70 (0.12)CD	0.80 (0.13)C	1.82 (0.36)B	2.50 (0.46)A

^*a*^Means within a life stage followed by the same letter are not significantly different based on Tukey’s HSD test (α = 0.05).

The weekly insecticide use regime was sprayed nine times at each location and each year. A similar number of sprays was needed for the nontraited and MON 88702 cotton for the early season only (*F* = 3.0; df = 1, 3; *P* = 0.18), late season only (*F* = 6.82; df = 1, 3; *P* = 0.08), and 2× threshold treatments (*F* = 1.42; df = 1, 3; *P* = 0.32). The mean (SEM) number of sprays were 2.00 (0.41) and 1.50 (0.29) for early season only; 3.50 (0.87) and 2.25 (0.85) for late season only; and 2.25 (0.95) and 1.50 (0.64) for the 2× threshold, for the nontraited and MON 88702, respectively. A difference in the number of sprays was observed for the treatment that was sprayed season long at the current action threshold (*F* = 27.0; df = 1, 3; *P* = 0.01). A mean (SEM) of 4.25 (0.85) and 2.75 (0.85) sprays were triggered based on the current action threshold in nontraited and MON 88702 cotton, respectively. Total tarnished plant bug numbers in the threshold treatment were reduced by 60.0 and 37.8% relative to the untreated control in MON 88702 and nontraited cotton, respectively (Table 3), suggesting that the insecticides worked better on MON 88702 despite being sprayed less often. Insecticides remain an important component of IPM programs for tarnished plant bug management in cotton. These data suggest that MON 88702 cotton expressing the Bt Cry51Aa2.834_16 protein may provide a complementary component to current IPM programs by reducing the number of tarnished plant bugs and the frequency of sprays needed to manage them in cotton.

### Cotton Yields

**Fig. 2.**
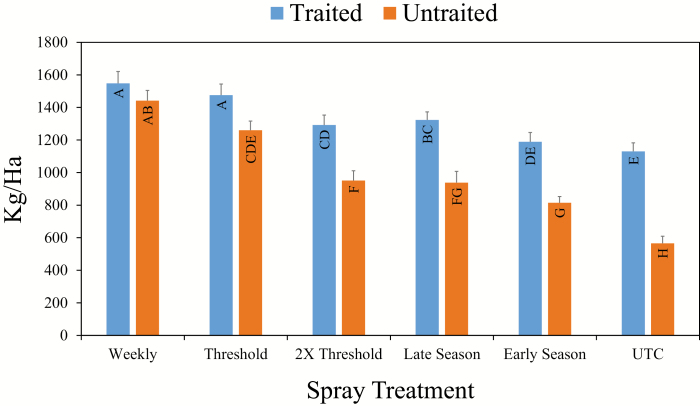
Impact of the interaction between cotton trait (Bt Cry51Aa2.834_16 vs conventional) and threshold spray treatment on cotton lint yields averaged across three site years in Mississippi during 2016 and 2017. The Threshold treatment was sprayed based on action thresholds published in the Mississippi Insect Control Guide for Agronomic Crops (Catchot et al. 2018) throughout the season. The 2× Threshold treatment was sprayed based on 2× action thresholds throughout the season. The Early Season treatment was sprayed based on current action thresholds from first square until first flower and left unsprayed the remainder of the season. The Late Season treatment was not sprayed from first square to first flower and was sprayed from first flower through cutout based on the current threshold.

For cotton yields, there was a trait by spray treatment interaction (*F* = 5.85; df = 5, 55; *P* < 0.01). No differences in yield were observed between the MON88702 cotton and cotton without the trait for the weekly insecticide use regime spray treatment (Fig. 2). In contrast, yields of the MON 88702 cotton were greater than the cotton without the trait for all other spray treatments. For the MON 88702 cotton, the current recommended thresholds throughout the different stages of cotton development appear to be appropriate, although threshold levels between 1× and 2× were not tested in this study. Yields were lower in the 2× threshold treatment compared to the threshold treatment for MON 88702 cotton. As a result, raising the threshold for MON 88702 cotton was not warranted based on these data. Additionally, yields of MON 88702 cotton in the threshold treatment were similar to yields in the weekly insecticide use regime of both cotton types, further supporting the current thresholds for tarnished plant bug in MON 88702 cotton. More research is needed to evaluate thresholds between the 1× and 2× thresholds evaluated in this study before definitive conclusions can be drawn regarding raising threshold levels for MON 88702. In cotton that was not sprayed for tarnished plant bug, yield of MON 88702 cotton was 1130 kg ha^-1^ compared to 565 kg ha^-1^ for cotton without the trait. Although these were not true isolines and there may be slight agronomic differences, yields of the two cotton types were similar in the weekly insecticide use regime. Because of that, these data do provide an indication of the potential yield benefit that can be observed with this trait in cotton. The Bt Cry51Aa2.834_16 trait in MON 88702 provided similar levels of protection during the early (preflower) and late season (flowering) stages of cotton development (Fig. 2).

Since the development of insecticide resistance in the mid-1990s to early-2000s (Snodgrass 1996, Snodgrass and Scott 2002, Snodgrass et al. 2009), there has been a renewed focus on integrated pest management (IPM) for tarnished plant bug in cotton. Experiments were conducted to evaluate and refine sampling procedures, economic injury levels, and action thresholds across the mid-southern United States (Musser et al. 2007, 2009a,b; Gore et al. 2012). Multiple agronomic practices have been evaluated in an overall IPM approach to manage tarnished plant bug in cotton as well. Planting date and cotton cultivar selection can impact tarnished plant bug management. In a previous study, a later maturing full season variety at later planting dates (after mid-May) required more insecticide applications compared to a short season cotton variety planted at earlier dates (Adams et al. 2013). Cotton varieties with dense pubescence also can experience lower numbers of tarnished plant bug and require fewer foliar sprays compared to a smooth leaf variety (Wood et al. 2017). Cotton fertility (Samples et al. 2019) and irrigation strategy (Wood et al. 2019) have also been shown to impact tarnished plant bug populations and management in cotton. The culmination of these IPM practices and the registration of sulfoxaflor (Transform 50WG, Corteva AgriScience, Indianapolis, IN), a novel insecticide, have led to a reduction of insecticide sprays in the Mississippi Delta region from an average of 7.5 in 2007 (Williams 2008) to 3.5 in 2017 (Cook 2018).

Overall, MON 88702 cotton that expresses the Bt Cry51Aa2.834_16 trait reduced the number of tarnished plant bugs and their injury in cotton, reduced the number of sprays needed based on the current action threshold, and improved cotton yields compared to cotton without the trait. Based on these data, this novel trait will provide an additional component for current IPM programs in cotton. Yields in untreated MON88702 cotton were lower than yields in MON 88702 that was sprayed weekly and sprayed based on the current action threshold. This suggests that insecticide sprays based on regular scouting and other IPM practices previously mentioned will be required to protect cotton yields and profits for growers. This result is consistent with that of Graham and Stewart (2018). The information provided by this research will be important for formulating Extension recommendations about the management of tarnished plant bug when varieties expressing the Bt Cry51Aa2.834_16 trait are commercially available, but more research is needed to accurately determine if the action threshold should be somewhere between the 1× and 2× thresholds tested.

## Supplementary Material

toaa075_suppl_Supplementary_Table_S1

toaa075_suppl_Supplementary_Table_S2

toaa075_suppl_Supplementary_Table_S3

toaa075_suppl_Supplementary_Table_S4
