# Supplementary material for: Evaluation of Current Tarnished Plant Bug (Hemiptera: Miridae) Thresholds in Transgenic MON 88702 Cotton Expressing the Bt Cry51Aa2.834_16 Trait
Source: J Econ Entomol. 2020 Apr 25;113(4):1816–22. doi: 10.1093/jee/toaa075 (PMC7425782; doi:10.1093/jee/toaa075)
Supplement: toaa075_suppl_Supplementary_Table_S1 [file toaa075_suppl_supplementary_table_s1.docx]

Supp. Table S1. Means and standard errors for tarnished plant bug, *Lygus lineolaris* (Palisot de Beauvois), numbers based on sweep net samples from first square to first flower of MON 88702 cotton expressing the Bt Cry51Aa2.834_16 protein and non-traited cotton in Sidon, MS and Stoneville, MS during 2016 and 2017. Means are expressed as numbers per 15 sweeps.

|  |  | Week 1 | |  | Week 2 | |  | Week 3 | |  | Week 4^1^ | |
| --- | --- | --- | --- | --- | --- | --- | --- | --- | --- | --- | --- | --- |
| Spray Treatment |  | MON 88702 | Non-Traited |  | MON 88702 | Non-Traited |  | MON 88702 | Non-Traited |  | MON 88702 | Non-Traited |
| Weekly |  | 0.8 (0.2) | 0.5 (0.1) |  | 0.6 (0.2) | 1.2 (0.3) |  | 0.7 (0.2) | 1. (0.2) |  | 1.3 (0.4) | 0.4 (0.2) |
| Threshold |  | 0.8 (0.2) | 0.3 (0.1) |  | 1.0 (0.2) | 1.4 (0.3) |  | 2.2 (0.7) | 1. (0.4) |  | 0.5 (0.3) | 1.9 (0.6) |
| 2X Threshold |  | 0.7 (0.2) | 1. (0.2) |  | 1.2 (0.3) | 1. (0.3) |  | 1.8 (0.4) | 2.3 (0.5) |  | 0.6 (0.3) | 1.8 (0.6) |
| Late Season Only |  | 0.7 (0.2) | 0.7 (0.2) |  | 1.8 (0.3) | 1.7 (0.3) |  | 1.8 (0.5) | 2.6 (0.5) |  | - 1. (0.5) | 2.5 (0.6) |
| Early Season Only |  | 0.6 (0.2) | 0.9 (0.2) |  | 1.5 (0.3) | 1.5 (0.3) |  | 1.7 (0.5) | 1.5 (0.3) |  | 1. (0.4) | 0.5 (0.2) |
| Untreated Control |  | 0.9 (0.2) | 1. (0.2) |  | 1.5 (0.3) | 2.3 (0.5) |  | 2.7 (0.6) | 2.2 (0.6) |  | - 1. (0.1) | 1.4 (0.3) |

^1^Means and standard errors for week four are based on one location in one year that had not started flowering by the fourth week of squaring.
