# Supplementary material for: Evaluation of Current Tarnished Plant Bug (Hemiptera: Miridae) Thresholds in Transgenic MON 88702 Cotton Expressing the Bt Cry51Aa2.834_16 Trait
Source: J Econ Entomol. 2020 Apr 25;113(4):1816–22. doi: 10.1093/jee/toaa075 (PMC7425782; doi:10.1093/jee/toaa075)
Supplement: toaa075_suppl_Supplementary_Table_S2 [file toaa075_suppl_supplementary_table_s2.docx]

Supp. Table S2. Means and standard errors for percent square retention during from first square to first flower of MON 88702 cotton expressing the Bt Cry51Aa2.834_16 protein and non-traited cotton in Sidon, MS and Stoneville, MS during 2016 and 2017.

|  |  | Week 1 | |  | Week 2 | |  | Week 3 | |
| --- | --- | --- | --- | --- | --- | --- | --- | --- | --- |
| Spray Treatment |  | MON 88702 | Non-Traited |  | MON 88702 | Non-Traited |  | MON 88702 | Non-Traited |
| Weekly |  | 88.8 (2.6) | 81.5 (3.4) |  | 92.3 (1.3) | 89.3 (2.0) |  | 90.0 (3.0) | 90.5 (1.9) |
| Threshold |  | 87.2 (3.4) | 78.0 (5.0) |  | 91.8 (1.2) | 87.6 (2.0) |  | 86.8 (3.3) | 86.3 (2.7) |
| 2X Threshold |  | 85.8 (3.2) | 75.8 (5.3) |  | 88.3 (2.6) | 84.3 (2.8) |  | 86.0 (2.9) | 83.6 (3.0) |
| Late Season Only |  | 88.0 (2.7) | 79.0 (4.3) |  | 83.3 (2.4) | 78.3 (2.8) |  | 84.3 (2.5) | 76.5 (2.8) |
| Early Season Only |  | 88.3 (2.4) | 79.8 (5.2) |  | 89.8 (2.1) | 83.0 (4.1) |  | 87.8 (2.2) | 88.0 (2.6) |
| Untreated Control |  | 86.0 (2.9) | 77.8 (3.5) |  | 84.8 (2.4) | 76.3 (3.4) |  | 84.5 (4.1) | 77.3 (2.9) |
