# Supplementary material for: Evaluation of Current Tarnished Plant Bug (Hemiptera: Miridae) Thresholds in Transgenic MON 88702 Cotton Expressing the Bt Cry51Aa2.834_16 Trait
Source: J Econ Entomol. 2020 Apr 25;113(4):1816–22. doi: 10.1093/jee/toaa075 (PMC7425782; doi:10.1093/jee/toaa075)
Supplement: toaa075_suppl_Supplementary_Table_S3 [file toaa075_suppl_supplementary_table_s3.docx]

Supp. Table S3. Means and standard errors for percentage of dirty (damaged) squares from first flower to cutout of MON 88702 cotton expressing the Bt Cry51Aa2.834_16 protein and non-traited cotton in Sidon, MS and Stoneville, MS during 2016 and 2017.

|  |  | WOF^1^ 1 | |  | WOF^1^ 2 | |  | WOF^1^ 3 | |
| --- | --- | --- | --- | --- | --- | --- | --- | --- | --- |
| Spray Treatment |  | MON 88702 | Non-Traited |  | MON 88702 | Non-Traited |  | MON 88702 | Non-Traited |
| Weekly |  | 1.5 (0.7) | 2.8 (1.1) |  | 1.0 (0.5) | 2.0 (0.6) |  | 2.0 (0.7) | 1.8 (0.6) |
| Threshold |  | 3.3 (0.9) | 2.3 (0.7) |  | 2.7 (0.8) | 3.0 (0.7) |  | 1.5 (0.6) | 2.0 (0.7) |
| 2X Threshold |  | 3.8 (0.9) | 4.5 (0.9) |  | 2.7 (0.9) | 4.7 (0.8) |  | 2.5 (1.0) | 4.3 (0.9) |
| Late Season Only |  | 3.8 (1.2) | 6.0 (1.3) |  | 2.0 (0.8) | 3.0 (0.7) |  | 3.0 (0.7) | 2.8 (0.7) |
| Early Season Only |  | 4.8 (1.7) | 4.0 (1.2) |  | 1.0 (0.5) | 3.7 (1.0) |  | 3.5 (1.1) | 4.0 (1.0) |
| Untreated Control |  | 3.5 (1.0) | 7.3 (1.5) |  | 1.0 (0.7) | 4.3 (1.7) |  | 5.5 (1.3) | 8.8 (2.0) |
|  |  |  |  |  |  |  |  |  |  |
|  |  | WOF^1^ 4 | |  | WOF^1^ 5 | |  | WOF^1^ 6 | |
| Spray Treatment |  | MON 88702 | Non-Traited |  | MON 88702 | Non-Traited |  | MON 88702 | Non-Traited |
| Weekly |  | 3.3 (1.0) | 3.8 (1.8) |  | 6.0 (1.3) | 4.5 (1.6) |  | 3.0 (1.9) | 2.0 (2.0) |
| Threshold |  | 5.0 (1.1) | 3.3 (1.1) |  | 8.5 (2.3) | 7.5 (2.9) |  | 6.0 (3.8) | 10.0 (7.6) |
| 2X Threshold |  | 5.5 (2.3) | 5.3 (1.7) |  | 12.0 (2.9) | 13.0 (2.6) |  | 21.0 (4.4) | 8.0 (5.7) |
| Late Season Only |  | 4.3 (1.2) | 4.5 (1.3) |  | 12.0 (4.5) | 7.0 (2.0) |  | 8.0 (3.3) | 6.0 (3.8) |
| Early Season Only |  | 3.0 (0.7) | 9.0 (1.5) |  | 14.0 (2.1) | 26.0 (3.9) |  | 18.0 (8.2 | 26.0 (10.0) |
| Untreated Control |  | 6.3 (0.7) | 11.5 (2.2) |  | 17.0 (4.3) | 16.5 (4.0) |  | 14.0 (7.7) | 24.0 (8.6) |

^1^Week of flowering
