# Supplementary material for: Evaluation of Current Tarnished Plant Bug (Hemiptera: Miridae) Thresholds in Transgenic MON 88702 Cotton Expressing the Bt Cry51Aa2.834_16 Trait
Source: J Econ Entomol. 2020 Apr 25;113(4):1816–22. doi: 10.1093/jee/toaa075 (PMC7425782; doi:10.1093/jee/toaa075)
Supplement: toaa075_suppl_Supplementary_Table_S4 [file toaa075_suppl_supplementary_table_s4.docx]

Supp. Table S4. Means and standard errors for tarnished plant bug, *Lygus lineolaris* (Palisot de Beauvois), numbers based on drop cloth samples from first flower to cutout of MON 88702 cotton expressing the Bt Cry51Aa2.834_16 protein and non-traited cotton in Sidon, MS and Stoneville, MS during 2016 and 2017. Means are expressed as number per two drop cloth samples (3.02-m of row).

|  |  | WOF^1^ 1 | |  | WOF^1^ 2 | |  | WOF^1^ 3 | |
| --- | --- | --- | --- | --- | --- | --- | --- | --- | --- |
| Spray Treatment |  | MON 88702 | Non-Traited |  | MON 88702 | Non-Traited |  | MON 88702 | Non-Traited |
| Weekly |  | 0.8 (0.5) | 0.9 (0.3) |  | 0.4 (0.2) | 1.2 (0.5) |  | 0.9 (0.3) | 2.2 (0.5) |
| Threshold |  | 1.0 (0.5) | 0.7 (0.3) |  | 2.0 (0.6) | 2.4 (0.7) |  | 2.9 (0.7) | 7.0 (2.1) |
| 2X Threshold |  | 3.6 (1.0) | 3.9 (1.4) |  | 3.8 (1.0) | 6.1 (1.7) |  | 8.7 (2.4) | 5.3 (1.1) |
| Late Season Only |  | 3.2 (0.7) | 5.6 (1.2) |  | 3.2 (1.1) | 5.0 (1.8) |  | 7.1 (2.5) | 4.0 (1.1) |
| Early Season Only |  | 0.8 (0.3) | 1.5 (0.4) |  | 2.3 (0.5) | 3.1 (1.0) |  | 11.3 (3.6) | 16.3 (4.7) |
| Untreated Control |  | 3.5 (1.0) | 4.7 (0.8) |  | 2.9 (0.7) | 6.6 (1.6) |  | 8.3 (2.9) | 16.4 (3.5) |
|  |  |  |  |  |  |  |  |  |  |
|  |  | WOF^1^ 4 | |  | WOF^1^ 5 | |  | WOF^1^ 6 | |
| Spray Treatment |  | MON 88702 | Non-Traited |  | MON 88702 | Non-Traited |  | MON 88702 | Non-Traited |
| Weekly |  | 0.4 (0.2) | 1.9 (0.5) |  | 1.3 (0.6) | 5.9 (1.8) |  | 3.1 (0.5) | 2.1 (0.6) |
| Threshold |  | 2.5 (0.4) | 4.3 (0.9) |  | 2.8 (1.0) | 3.8 (.7) |  | 4.5 (1.2) | 34.4 (15.4) |
| 2X Threshold |  | 3.7 (0.7) | 4.6 (0.8) |  | 7.5 (1.6) | 11.3 (2.6) |  | 15.3 (3.1) | 19.8 (6.6) |
| Late Season Only |  | 2.4 (0.5) | 2.9 (0.7) |  | 7.3 (2.0) | 7.0 (2.4) |  | 3.1 (1.7) | 2.1 (4.1) |
| Early Season Only |  | 6.9 (1.2) | 8.8 (1.5) |  | 6.6 (1.8) | 11.6 (3.3) |  | 6.1 (1.9) | 11.5 (1.7) |
| Untreated Control |  | 7.3 (1.5) | 9.9 (1.4) |  | 6.1 (2.0) | 11.4 (2.8) |  | 7.5 (2.2) | 13.4 (2.4) |

^1^Week of flowering
